# Vesicle miR-195 derived from Endothelial Cells Inhibits Expression of Serotonin Transporter in Vessel Smooth Muscle Cells

**DOI:** 10.1038/srep43546

**Published:** 2017-03-08

**Authors:** Junzhong Gu, Huiyuan Zhang, Bingyang Ji, Hui Jiang, Tao Zhao, Rongcai Jiang, Zhiren Zhang, Shengjiang Tan, Asif Ahmed, Yuchun Gu

**Affiliations:** 1 Molecular Pharmacology Laboratory, Institute of Molecular Medicine, Peking University, Beijing, China; 2 Department of Cardiopulmonary Bypass, Cardiovascular Institute and Fuwai Hospital, PUMC & CAMS, Beijing, China; 3 Beijing Key Laboratory of Reproductive Endocrinology and Assisted Reproduction, Department of Urology, Peking University Third Hospital, Beijing, China; 4 Suzhou University, Suzhou, China; 5 Tianjin Neurological Institute, Tianjin 300052, China; 6 Key Laboratory of Post-Neuroinjury Repair and Regeneration in Central Nervous System, Ministry of Education and Tianjin City, Tianjin 300052, China; 7 Department of Pharmacology, 2nd affiliated hospital of Harbin Medical University, Harbin, China; 8 Cambridge Institute for Medical Research, Cambridge, UK; 9 Department of Haematology, University of Cambridge, Cambridge, UK; 10 Wellcome Trust-Medical Research Council Stem Cell Institute, University of Cambridge, Cambridge, UK; 11 Aston Medical School, Aston University, Birmingham, UK; 12 Novolife Translational and Regenerative Medicine Centre, Aston Medical School, Aston University, Birmingham, UK

## Abstract

Serotonin or 5-hydroxytryptamine (5-HT) has been shown to be essential in lots of physiological and pathological processes. It is well known that 5-HT and 5-HT transporter (5-HTT) play important roles in the pulmonary artery in pulmonary hypertension. However, little is known about the function of 5-HTT in other arteries. In this study we found that the expression of 5-HTT was elevated in injured carotid arteries and over-expression of 5-HTT induced proliferation of smooth muscle cells (SMCs); however, this phenotype could be reversed by knocking-down of 5-HTT or endothelial cells conditional medium (EC-CM). A 5-HTT inhibitor, fluoxetine, treated animals also exhibited reduced restenosis after injury. We identified that miR-195 was packaged in the extracellular vesicles from EC-CM. We further confirmed that extracellular vesicles could transfer miR-195 from ECs to SMCs to inhibit the expression of 5-HTT in SMCs and the proliferation of SMCs. These results provide the first evidence that ECs communicate with SMCs via micro-RNA195 in the regulation of the proliferation of SMCs through 5-HTT, which will contribute to a better understanding of communications between ECs and SMCs via micro-RNA. Our findings suggest a potential target for the control of vessel restenosis.

Serotonin or 5-hydroxytryptamine (5-HT) is a monoamine neurotransmitter and is widely present in organisms from nematodes to human^1^. 5-HT is associated with depression^2^, suicidal behavior and other mental disorders^3^. Apart from its function in CNS, the role of 5-HT in the cardiovascular system also attracts more and more attention. It is now well known that serotonin stimulates smooth muscle cell growth and migration^4^,^5^. 5-HT and serotonin transporter (5-HTT) play a critical role in pulmonary arterial hypertension (PAH) pathogenesis. Inhibition of 5-HTT can prevent and reverse PAH^6^. Activation of 5-HT2B receptors also regulates the development of pulmonary hypertension^7^. Proliferation and apoptosis of vascular smooth muscle cells (SMCs) have a critical role in vascular neointimal lesion formation and atherosclerosis^8^. Although SMCs in adults blood vessels are quiescent, they can be activated and become highly proliferative during vascular injury^9^. Several studies have shown that the communication between ECs and SMCs is important to regulate SMCs homeostasis^10^,^11^,^12^. However, the mechanism underlying such phenomenon remains unclear.

MicroRNAs (miRNAs) are endogenous noncoding RNAs which approximately consist of 22 nucleotides. MicroRNAs can bind to mRNAs, which leads to the degradation or translational repression of its targeted mRNAs to inhibit gene expression. MicroRNAs have been proved to be messengers that can get into recipient cells to regulate gene expression^10^,^12^,^13^,^14^. Apart from their intrinsic cellular function, the circulating microRNAs are enticing biomarkers and potential therapeutic targets for cardiovascular diseases^15^,^16^. ECs and SMCs play critical roles in vasculature homeostasis. MicroRNAs have a key role in regulating ECs function^17^ and SMCs turnover^18^. MicroRNA-195 has been demonstrated in association with cardiac hypertrophy, heart failure^19^ and diabetic cardiomyopathy^20^. In this study, we addressed how miR-195 is involved in the homeostasis of vessel smooth muscle cells.

## Results

### Inhibition of Serotonin transporter (5-HTT) prevents neointima formation after balloon injury

**Figure 1:**
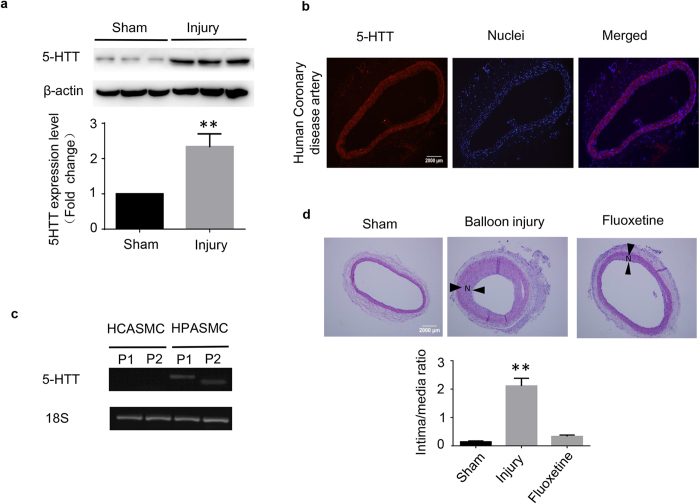
Serotonin transporter (5-HTT) inhibition prevents neointima formation after balloon injury. (**a**) The protein level of 5-HTT in the injury and the normal carotid arteries from rat was measured by WB. Images are representative of triplicate experiments with similar results. Full-length blots are presented in Supplementary Figure 1a. **P < 0.01 compared with the sham (n = 3). (**b**) The expression of 5-HTT in coronary tissue (red) was detected by immunostaining. Nuclei were stained with DAPI (blue). Scale bar, 2000 μm. (**c**) RT-PCR was used to detect the expression of 5-HTT in human coronary artery smooth muscle cell (HCASMCs) and human pulmonary artery smooth muscle cell (HPASMCs). Full-length gels are presented in Supplementary Figure 1b. P1: primer 1, product = 162 bp; P2: primer2, product = 98 bp. (**d**) Representative images of balloon injured carotid arteries treated with vehicle or fluoxetine (n = 8). 2 weeks after the injury, the arteries were fixed followed with sectioning and staining with hematoxylin and eosin. Intima/media ratios were calculated from at least 6 rats per group. **P < 0.01 compared with sham arteries. N: neointima. Scale bar, 2000 μm.

5-HTT plays a critical role in pulmonary arterial hypertension (PAH) pathogenesis. To identify the role of 5-HTT in injury stimulated vascular remodeling, we performed balloon angioplasty injury on rat carotid arteries and examined the expression of 5-HTT. 14 days after injury, the expression of 5-HTT increased approximately 2-fold in the injured artery compared to the contralateral uninjured carotid artery (Fig. 1a). To address whether 5-HTT is expressed in other arteries, human coronary arteries obtained from coronary disease patients were analyzed by immunohistostaining. Abundant expression of 5-HTT was found in the patient’s coronary artery (Fig. 1b). Noticeably, the expression of 5-HTT was detected in the cultured human pulmonary smooth muscle cells (HPASMCs), but not in normal human coronary smooth muscle cells (HCASMCs) as revealed by RT-PCR (Fig. 1c). After fluoxetine, a 5-HTT inhibitor, was applied to rats to assess the potential preventive effect of 5-HTT suppression on neointima formation, the neointima formation was markedly attenuated in fluoxetine-treated rats compared with the control group (Fig. 1d). Together, these results indicate that 5-HTT is involved in the neointima formation of injured artery.

### Over-expression of 5-HTT promotes the proliferation of SMCs

**Figure 2:**
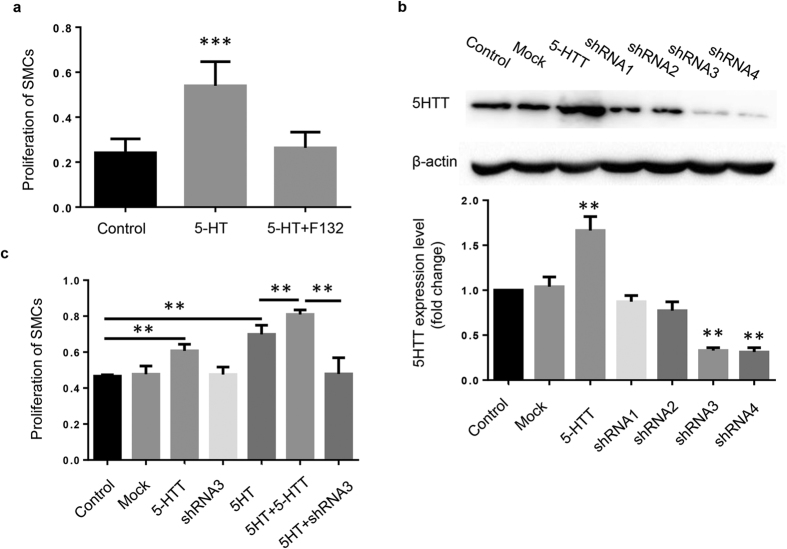
5-HTT promotes SMCs proliferation. (**a**) SMCs were treated with 5-HT or 5-HTT inhibitor F132. The proliferation of SMCs was determined by MTT. **P < 0.01 compared with control (n = 6). (**b**) Western blot analysis confirmed the effects of 5-HTT overexpression and shRNA knockdown on the protein levels compared to scrambled controls. Full-length blots are presented in Supplementary Figure 1c. **P < 0.01 compared with scramble controls (n = 3). (**c**)The proliferation level of HUVSMCs after 5-HTT overexpression and knockdown was measured by MTT. **P < 0.01 compared with control (n = 6).

The proliferation of SMCs is the major event during the neointimal formation. To determine whether 5-HTT is related to the proliferation of SMCs, the effects of 5-HTT on serum-stimulated cell growth of VSM were examined in human umbilical vein SMC freshly isolated from human umbilical cord vein. 5-HT (10 μM) was found to promote the proliferation of SMCs, but this effect could be reversed by a 5-HTT inhibitor, F132 (1 μM) (Fig. 2a). The over-expression and shRNA knockdown of 5-HTT were confirmed by immunoblotting (Fig. 2b). Overexpression of 5-HTT promoted the proliferation of SMCs and the effect of 5HT was mildly enhanced when 5-HTT was over-expressed. Furthermore, knockdown of 5-HTT by shRNA inhibited the proliferation of SMCs (Fig. 2c). Taken together, these results suggest that 5-HTT could stimulate the proliferation of SMCs.

### ECs conditional medium (EC-CM) inhibits the expression of 5-HTT in SMCs and the proliferation of SMCs

**Figure 3:**
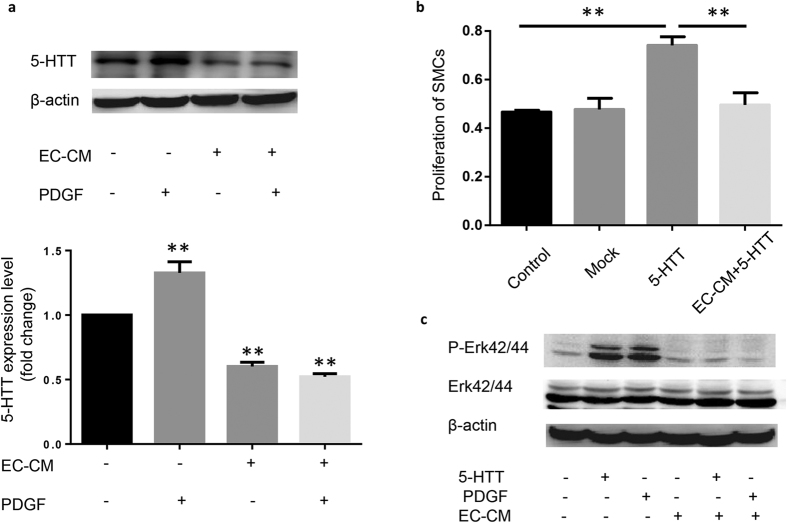
ECs regulate the 5-HTT expression and proliferation of SMCs. (**a**) SMCs was cultured with EC-CM and the protein level of 5-HTT was analyzed by Western blot 48 hours later. Full-length blots are presented in Supplementary Figure 2a. **P < 0.01 compared with control (n = 3). (**b**) The proliferation level of SMCs after treated with EC-CM was measured by MTT. **P < 0.01 compared with control (n = 6). (**c**) SMCs were incubated with EC-CM or PDGF for 48 hours. Phosphorylation of ERK was determined by Western blot analysis of the whole cell lysate using the phosphor-specific antibody. Full-length blots are presented in Supplementary Figure 2b.

SMCs can be activated to be proliferative under certain pathophysiological conditions such as injury of ECs. We found that the communication between ECs and SMCs could regulate the expression of 5-HTT and the proliferation of SMCs. ECs were cultured in growth medium for 48 hours and the ECs conditional medium (EC-CM) was collected. SMCs were cultured in EC-CM and D/F12 medium (1:1) for 48 hours. Then the 5-HTT in SMC was detected by immunoblotting. We found that the expression of 5-HTT in SMCs was suppressed by EC-CM (Fig. 3a). EC-CM could also inhibit the proliferation of SMCs induced by the overexpression of 5-HTT (Fig. 3b). Furthermore, PDGF-induced upregulation of 5-HTT was also inhibited by EC-CM (Fig. 3a). Previous studies have shown that 5-HTT-initiated Rho kinase signaling elicited 5-HTR1B mediated phosphorylation of ERK by MEK. Translocation of pERK to the nucleus activated transcription factors then promoted calcium dependent proliferation in the PASMCs^21^,^22^. Similarly, we found elevated phosphorylation of Erk42/44 induced by overexpression of 5-HTT and PDGF. The elevated phosphorylation of Erk42/44 was inhibited by EC-CM (Fig. 3c). These results demonstrate that the communication between ECs and SMCs could regulate the expression of 5-HTT and the proliferation of SMCs.

### Vesicles derived from ECs modulates the expression of 5-HTT in SMCs

**Figure 4:**
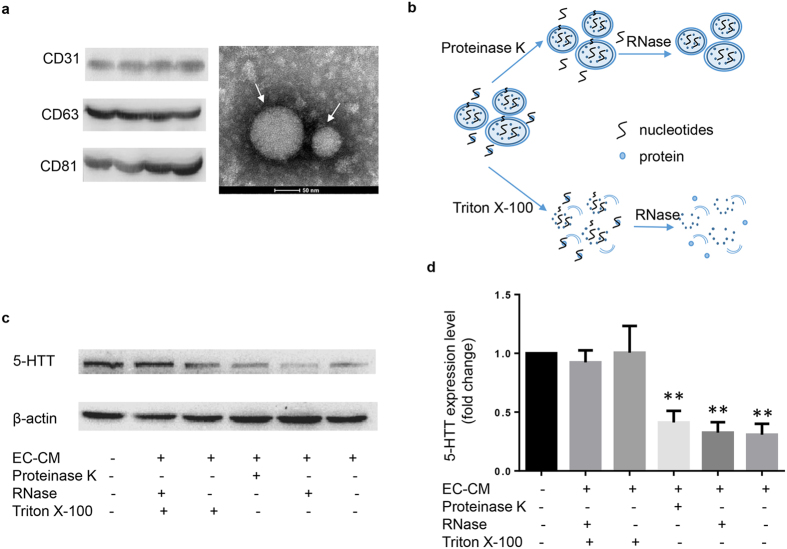
EC-secreted vesicles regulate SMCs 5-HTT gene expression. (**a**) Left: western blot analysis of exosome protein. Full-length blots are presented in Supplementary Figure 2c. Right: Electron microscopy image of isolated extracellular vesicles of ECs (30–100 nm). (**b**) Schematic of the degradation assay used in this study. (**c**) EC-CM was incubated with the indicated reagents cultured SMCs. The protein of SMCs was extracted 48 hours later and the levels of 5-HTT was measured by WB. Full-length blots are presented in Supplementary Figure 3a. (**d**)Semi-quantification results of 5-HTT protein level after cultured with different treated EC-CM. **P < 0.01 compared with control (n = 3).

As EC-CM could regulate the expression of 5-HTT in SMCs we speculated that EC-CM might affect the function of SMCs in a paracrine manner. Several recent studies indicate that vesicles can transduce signals between cells^10^,^23^,^24^. To study the effect of EC-CM on the expression of 5-HTT in SMCs, we isolated vesicles from EC-CM and identified the isolated vesicles using electron microscopy (Fig. 4a). The diameter of the vesicles was between 30 and 100 nm, indicating that most of the isolated vesicles were comprised of exosomes rather than larger apoptotic bodies. The isolated exosome fractions were immunoreactive for the exosomal marker CD63 and CD81. In addition, CD31 was also detected in the isolated exosomes, indicating that the isolated exosomes were secreted by ECs. To further identify the function of exosomes isolated from EC-CM, we investigated the mode of alteration in EC-CM upon different treatments (Fig. 4b). We found that protein degradation by proteinase K or nucleotides degradation by RNase did not affect the function of EC-CM (Fig. 4c). In contrast, treatment with phospholipid membrane disruptors, Triton X-100, before RNase treatment led to a dramatic abolishment of the function of EC-CM, which indicates that extracellular vesicles in EC-CM were regulators of the expression of 5-HTT in SMCs.

### Extracellular vesicles transfer miR195 from ECs to SMCs

**Figure 5:**
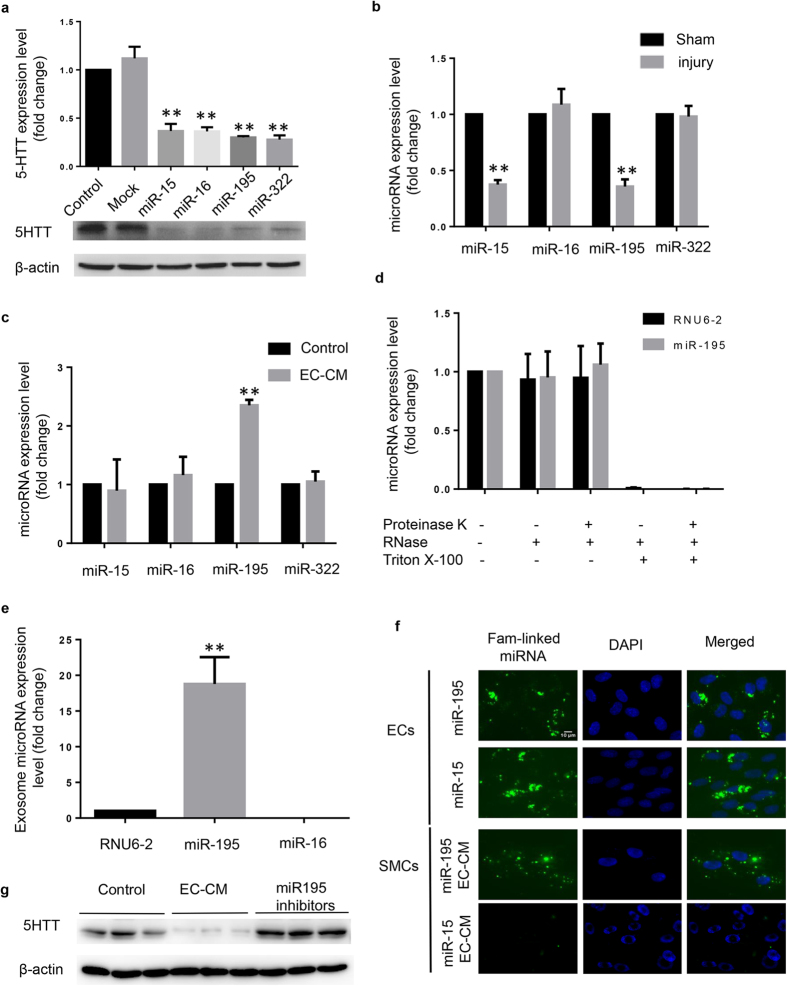
Endothelial cells transmit miR-195 to SMCs via vesicles. (**a**) 5-HTT expression was inhibited after miR-15, miR-195, miR16, miR-322 mimics transfection in SMCs. The micro-RNA mimics were transfected into SMCs and the protein was extracted after 48 hours. Full-length blots are presented in Supplementary Figure 3b. **P < 0.01 compared with control (n = 3). (**b**) The miRNA level of miR-15 and miR-195 were measured by real-time PCR in normal and injured carotid artery. **P < 0.01 compared with shame (n = 3). (**c**) SMCs was cultured by EC-CM 24 hours when RNA was isolated. The miRNA level was detected by real time PCR. **P < 0.01 compared with control (n = 4). (**d**) The miRNAs expression level of extracellular vesicles from ECs was measured by real-time PCR (n = 3). (**e**) Extracellular vesicles of ECs were incubated with the indicated reagents before isolating RNA and measuring the levels of the indicated miRNAs by real-time PCR. **P < 0.01 compared with control (n = 4). (**f**) SMCs were treated with EC-CM from FAM-linked miRNA transfected ECs. FAM-linked miRNA in ECs and SMCs was detected by fluorescence microscopy. (**g**) ECs were transfected with miR-195 inhibitors and the EC-CM were used to culture SMCs. The protein of SMCs was extracted 48 hours later and the levels of 5-HTT was measured by WB. Full-length blots are presented in Supplementary Figure 3c.

We hypothesized that it was microRNAs in EC-CM vesicles that regulated the expression of 5-HTT. According to the prediction of TARGETSCAN (http://www.targetscan.org/), we selected miR-15, miR-16, miR-195 and miR-322 as candidates to test their function associated with the expression of 5-HTT in SMC. After SMCs were transfected with miRNA mimics we found that miR-15, miR-16, miR-195 and miR-322 could reduce the expression of 5-HTT (Fig. 5a). Next, we checked the expression of miR15, miR-16, miR-195 and miR-322 in injured carotid arteries and SMCs cultured in EC-CM. However, only miR-15 and miR-195 showed decreased expression in the balloon injured carotid arteries (Fig. 5b). In the EC-CM treated SMCs, only the expression of miR-195 increased (Fig. 5c). By using the mode of different treatments with EC-CM (Fig. 4b), we found that treatments with proteinase K or RNase did not affect expression of miR-195 (Fig. 5d). In contrast, treatment with phospholipid membrane disruptors Triton X-100 followed by RNase treatment led to an almost complete degradation of miR-195, indicating that endothelial-derived miR-195 was released and protected by extracellular vesicles. The expression of miRNAs in extracellular vesicles was measured by real-time quantitative PCR. The expression of miR-195 was approximately 19-fold high of the control, RNU6–2. The miR-15 expression was almost undetectable (Fig. 5e). To determine whether miR-195 and miR-15 were directly delivered into SMCs, synthetic miR-15 and miR-195 mimics (oligomer labeled with FAM) were transfected into ECs. The culture medium was replaced after 8 hours to remove the free FAM-labeled miRNAs. After 24 hours the EC-CM was collected for the incubation of SMCs. We found that EC-CM derived from ECs transfected with the FAM-miR195 led to the presence of FAM-miR195 in SMCs, but there is no presence of FAM-miR15 in SMCs after incubation of EC-CM derived from ECs transfected with the FAM-miR15 (Fig. 5f). To further confirm the protective effect of EC vesicles, ECs were transfected with miR-195 inhibitors. Then the EC-CM was collected to culture SMCs. We found that the miR-195 inhibitor transfected EC-CM could not reduce the expression of 5-HTT in SMCs (Fig. 5g). These results suggest a direct transmission of miR-195 from ECs to SMCs via vesicles derived from ECs.

## Discussion

5-HT and serotonin transporter (5-HTT) play important roles in the progression of pulmonary hypertension^6^,^11^,^25^. Although over-expression of 5-HTT promotes PAH, the functions of 5-HTT in other arteries remain unclear. In the present study, we demonstrated that the expression of 5-HTT was up-regulated in injured carotid arteries and over-expression of 5-HTT could induce proliferation of smooth muscle cells (SMCs) proliferation. Neointima formation is significant at two weeks and reaches it’s maximum at four weeks after balloon injury^16^,^26^. Similarly, we observed efficient neointima formation at two weeks after balloon injury. We also found that the expression of 5-HTT was up-regulated in the injured arteries. However, the neointima formation was markedly attenuated when the rats received fluoxetine. Although the present study only provides results from animals of two weeks after balloon injury, it still strongly suggests that 5-HTT plays an important role in neointima formation.

The excessive expression of 5-HTT accelerates the proliferation of SMCs. However, the regulation of the expression of 5-HTT is unknown in SMCs. Several studies have indicated that miR-15 and miR-16 regulate 5-HTT expression in brain and placental^27^,^28^. In the present study, we found that miR15, miR-16, miR-195 and miR-322 could reduce the expression of 5-HTT in SMCs. However, only the expression of miR-15 and miR-195 is decreased in the balloon injured carotid arteries. Furthermore, we found that only miR-195 in SMCs increased after incubation with EC-CM. These results indicate that miR-195 is a new regulator of 5-HTT in the blood vessels.

The elevated expression of miR-195 in SMCs after EC-CM treatment indicates that miRNAs can mediate signals between ECs and SMCs. Several studies have shown that the communication between cells is important to modulate cell function^10^,^11^,^29^,^30^,^31^. After binding to recipient cells, there are three fates of the extracellular vesicles (EVs): a. EVs may remain stably associated with the membrane^32^; b. EVs directly fuse with the membrane and release their contents^33^; c. EVs can be internalized through distinct endocytic pathways. When endocytosed, EVs may subsequently fuse with the endosomal delimiting membrane or be targeted to lysosomes for degradation^34^,^35^,^36^. To confirm that ECs could transfer functional miR-195 to SMCs, we transfected ECs with FAM-labeled miR-195 mimics. We found that ECs transfected with the FAM-miR195 led to the presence of FAM-miR195 in SMCs. When ECs were transfected with miR-195 inhibitors or EC-CM treated with Triton-100, EC-CM could not inhibit the expression of 5-HTT in SMCs. These data indicate that ECs derived vesicles can transfer miR-195 from ECs into SMCs to suppress the expression 5-HTT in SMCs. MicroRNAs have crucial roles in the regulation of gene expression and are involved in physiological and pathological processes, such as lipid metabolism and atherosclerosis. Variation in the expression of one miRNA can affect various steps of one signaling pathway^37^. Therefore miRNAs can be exploited as future diagnostic and even therapeutic targets. In the present study, we found that a new extracellular messenger derived from ECs, miR-195 could regulate the expression of 5-HTT in SMCs and the proliferation of SMCs. Thus miR-195/5-HTT may act as a promising therapeutic target for vascular restenosis in the future. Further investigation of the regulation of miR-195 in ECs will be eminent to evaluate its role in pathological processes, such as vessel restenosis. Further study is also needed to assess the protective effect of ECs on neointima formation, especially the effect of ECs from miR-195 knockout mice.

In summary, our data show that the overexpression of 5-HTT promotes the proliferation of SMCs and inhibition of 5-HTT prevents neointima formation in rat’s carotid artery. ECs derived miR-195 acts as a new regulator to modulate the expression of 5-HTT in SMCs. These findings confirm that ECs play an important role in the prevention of SMC from over-proliferation, which provides a new underlying mechanism responsible for the occurrence and development of restenosis.

## Material and Methods

### Cell culture and transfection

Human coronary and pulmonary smooth muscle cells (HCASMCs and HPASMCs) were purchased from Promo Cell. Human umbilical vein endothelial cells (HUVEC) and human umbilical vein smooth muscle cells (HUVSMCs) were freshly isolated from human umbilical cord vein as previously described^38^,^39^ and cultured with ECs growth medium (Promo Cell) and DMEM/F12 supplemented with 10% FCS, 1% sodium pyruvate, 1% glutamine and 1% penicillin and streptomycin respectively. The protocols for human umbilical cord collection and isolation of human umbilical vein endothelial cells (HUVECs) and human umbilical vein smooth muscle cells (HUVSMCs) were approved by University Ethics Committee, Institute of Molecular Medicine, Peking University. All methods for human tissues were performed in accordance with the relevant guidelines and regulations. The informed consent was obtained from all subjects.

The short hairpin RNA (shRNA) used in this study was obtained from Zhang laboratory, School of Life Sciences, Peking University, Beijing, China. NON-TARGET shRNA Control Vector was used as negative control in the 5-HTT knockdown experiment. The plasmid of 5-HTT over-expression was purchased from Vigene bioscience (Shandong, China). MicroRNA mimics and inhibitors were purchased from GenePharma (Shanghai, China). The plasmid, mircoRNA mimics and inhibitors were introduced into ECs and SMCs using Lipofectamine 2000 (Invitrogen) according to the manufacturer’s instructions.

### Animals

All experimental protocols were approved by University Ethics Committee, Institute of Molecular Medicine, Peking University. All animals received humane care in compliance Guide for the Institutional Animal Care and Use Committee (IACUC).

Adult male Sprague Dawley rats (300 g) were purchased from Vital River (Beijing, China). All surgeries were performed under sodium pentobarbital anesthesia, and all efforts were made to minimize suffering. Male Sprague-Dawley rats weighing 350–400 g were used in the balloon injury model. The rats were anesthetized via intraperitoneal injection with 2% sodium pentobarbital (Sigma). The rats were injected with 100U/kg heparinization before balloon injury. The left carotid arteries of the animals were isolated, and the balloon was introduced into the left common carotid through the left external carotid artery. The balloon was inflated to produce moderate resistance to catheter movement and then was withdrawn gradually to the near entry point. The entire procedure was repeated three times. Rats in sham group underwent the same procedure, except for artery injury. After the surgery, the rats were injected with penicillin (200,000 U) to prevent infections. Then, to assess the potential preventive effect of 5-HTT inhibition on neointima formation, the rats were assigned into two groups randomly (8 animals in each group): one group received fluoxetine at 10 mg/kg/day and one group received vehicle only. All animals were fed with conventional diet until sacrificed. Euthanasia was achieved by isoflurane inhalational anesthesia (1.5%) and concurrent an aesthetic overdose as approved by the respective University IACUCs.

### RNA preparation, RT-PCR and Real-time PCR analysis

Total RNA was isolated using TRIZOL (Invitrogen). Total RNA was reverse transcribed into cDNA using EasyScript First-Strand cDNA Synthesis SuperMix kit (TransGen Biotech). The cDNA used in real time PCR was synthesized by miRNA cDNA synthesis kit (CWBIO). The miRNA expression level was analyzed by Fast 7500 thermocycler (Applied Biosytems). RNU6-2 was used as a normalization control in all miRNA measurements. Primers used in this study are listed below.

5-HTT Primer 1:

F: 5-AGAATTTTACACGCGCCACG-3′; R:5′-GAGGTCTTGACGCCTTTCCA-3′

5-HTT Primer 2:

F:5- CCTCATTGCCACCTTCCTGT-3′; R:' 5- CAGCCAAGCCATGGTGACTA-3′

18S Primer:

F: 5′-CAGCCACCCGAGATTGAGCA-3′; R:5′-TAGTAGCGACGGGCGGTGT-3′

RNU6-2 Primer:

F: 5′- CTCGCTTCGGCAGCACA -3′; R:5′- AACGCTTCACGAATTTGCGT -3′

### Immunoblotting

The protein samples obtained from tissue or cell extracts were quantified using the Bradford reagent (BioRad). Before loading into the 10% SDS-PAGE, the samples were mixed with 5x loading buffer and boiled at 98 °C for 5 mins. After gel electrophoresis, the proteins were then transferred to a PVDF membrane (0.45 μm, Merck Millipore) under 250 mA for 90 mins. The membranes were incubated with a blocking buffer (5% nonfat milk in Tris-buffered saline containing 0.5% Tween-20) for 1hr at room temperature and then incubated with the indicated primary antibodies at 4 °C overnight. The membrane was incubated with horseradish peroxidase-conjugated goat anti-mouse immunoglobulin G (IgG; 1:5000) or with goat anti-rabbit IgG (1:5000) for one hour at room temperature. The target protein bands were visualized using Amersham Imager 600 (GE healthcare). The following antibodies were used: goat Anti-Serotonin transporter (Abcam, #ab130130), rabbit anti-p44/42 MAPK (Cell Signaling, #4695 s), rabbit anti-Phospho-p44/42 MAPK (Cell Signaling, #4370 s), mouse anti-ß-actin (Beijing TDY Biotech LTD, #M009), HRP-conjugated secondary mouse (Beijing TDY Biotech LTD, #E009) goat (Beijing TDY Biotech LTD, S008) and rabbit antibodies (Beijing TDY Biotech LTD, #E011).

### Cell proliferation assay

3,000 smooth muscle cells were seeded in each well of 96-well plate. The VSMCs were treated with DMEM/F12 including 0.5% FBS for 12 hours before the assay. After different treatments, 20 ul of MTT was added to each well for 3 h incubation. Subsequently, cells were dissolved by 150 mL of DMSO, mixed and measured the absorbance by Multiskan (Thermo) at 540 nm. The relative proliferation rate was calculated by the absorbance ratio of the drug-treated group to the control group.

### Extracellular vesicles isolation

Exosomes were isolated from conditioned medium (CM) using a multi-step centrifugation procedure as described previously^40^. Briefly, ECs were cultured with EC growth medium with exosome-free serum (Promo Cell). After cultivation for 72 h, the supernatant was collected centrifuge at 300 g for 10 min at 4 °C to pellet the cells. To remove cells and cell debris, the supernatant was centrifuged at 16,500 g for 20 min at 4 °C. Then filter the supernatant through a 0.2 μm filter to remove particles larger than 200 nm. The filtered supernatant was centrifuged at 120,00 g for 70 min at 4 °C to pellet the exosomes. The supernatant was discarded and the pelleted vesicles were re-suspended in a small volume of an appropriate buffer. 500 μl TRIZOL (Invitrogen) for RNA isolation or in PBS for electron microscopy.

### Electron microscopy

Exosomes were collected from EC-CM as described above, pelleted for preparation of the carbon coated copper grid (Beijing Zhongjingkeyi Technology). The grids with exosomes were negatively stained with 2% phosphotungstic acid (PTA), and imaged with transmission electron microscope.

### Histological analysis

The human coronary disease artery was obtained from Fuwai Hospital in Beijing, China. The collection of human artery tissue were performed in agreement with the guidelines approved by the Institutional Review Board at FuWai Hospital. All experimental protocols were approved by University Ethics Committee, Institute of Molecular Medicine, Peking University. All methods were performed in accordance with the relevant guidelines and regulations. Informed consents were obtained from all subjects. The artery was fixed in 4% paraformaldehyde and embedded in paraffin. For 5-HTT immunohistochemical staining, paraffin-embedded tissue sections were deparaffinized and rehydrated, and then autoclaved in 10 mmol/L citrate buffer for antigen retrieval. After quenching endogenous peroxidase, the sections were incubated with goat Anti-Serotonin transporter (Abcam, #ab130130) at 4 °C overnight. The sections were incubated with horseradish Cy3-conjugated goat anti-mouse IgG (IgG; 1:5000) for one hour at room temperature.

In the rat experiment, the left and right common arteries were isolated and fixed in 4% paraformaldehyde at the 14th day after operation. After embedded in paraffin, arteries were cut into sections of 4 μm thickness and stained with hematoxylin and eosin. The sections were viewed with an Eclipse 600 Nikon microscope and photographed with a digital camera.

### Statistical analysis

All statistical analyses were carried out using Graphpad Prism 6. Data are expressed as average ± SEM of at least three independent experiments. The statistical significance was determined using a t-test when comparing two groups and ANOVA when comparing multiple groups. A value of P < 0.05 was considered statistically significant.

## Additional Information

**How to cite this article:** Gu, J. *et al*. Vesicle miR-195 derived from Endothelial Cells Inhibits Expression of Serotonin Transporter in Vessel Smooth Muscle Cells. *Sci. Rep.* **7**, 43546; doi: 10.1038/srep43546 (2017).

**Publisher's note:** Springer Nature remains neutral with regard to jurisdictional claims in published maps and institutional affiliations.

## Supplementary Material

Supplementary Information
