# Supplementary material for: Vesicle miR-195 derived from Endothelial Cells Inhibits Expression of Serotonin Transporter in Vessel Smooth Muscle Cells
Source: Sci Rep. 2017 Mar 8;7:43546. doi: 10.1038/srep43546 (PMC5341127; doi:10.1038/srep43546)
Supplement: Supplementary Information [file srep43546-s1.pdf]

Vesicle miR-195 derived from Endothelial Cells Inhibits Expression of Serotonin Transporter in Vessel Smooth Muscle Cells

Junzhong Gu[1], Huiyuan Zhang[1], Bingyang Ji[2], Hui Jiang[3], Tao Zhao[4], Rongcai Jiang[5,6], Zhiren Zhang[7], Shengjiang Tan[8,9,10], Asif Ahmed[11] and Yuchun Gu[1,12*]

[1]Molecular Pharmacology Laboratory, Institute of Molecular Medicine, Peking University, Beijing, China

[2]Department of Cardiopulmonary Bypass, Cardiovascular Institute and Fuwai Hospital, PUMC & CAMS, Beijing, China.

[3]Beijing Key Laboratory of Reproductive Endocrinology and Assisted Reproduction, Department of Urology, Peking University Third Hospital, Beijing, China

[4]Suzhou University, Suzhou, China

[5]Tianjin Neurological Institute, Tianjin 300052, China.

[6]Key Laboratory of Post-Neuroinjury Repair and Regeneration in Central Nervous System, Ministry of Education and Tianjin City, Tianjin 300052, China.

[7]Department of Pharmacology, 2nd affiliated hospital of Harbin Medical University, Harbin, China

[8]Cambridge Institute for Medical Research, Cambridge, UK.

[9]Department of Haematology, University of Cambridge, Cambridge, UK.

[10]Wellcome Trust-Medical Research Council Stem Cell Institute, University of Cambridge, Cambridge, UK.

[11]Aston Medical School, Aston University, Birmingham, UK.

[12]Novolife Translational and Regenerative Medicine Centre, Aston Medical School, Aston University, Birmingham, UK.

Address for correspondence:
Yuchun Gu, MD, PhD
Chair in Molecular Pharmacology Laboratory,
Institute of Molecular Medicine (IMM), Peking University,
Room 216, Pacific Building, 52 Haidian Road
100871, Beijing, China
Tel: +86.10.8254.5679
Fax: +86.10.8257.5672
Email: ycgu@pku.edu.cn

Supplementary Figure 1

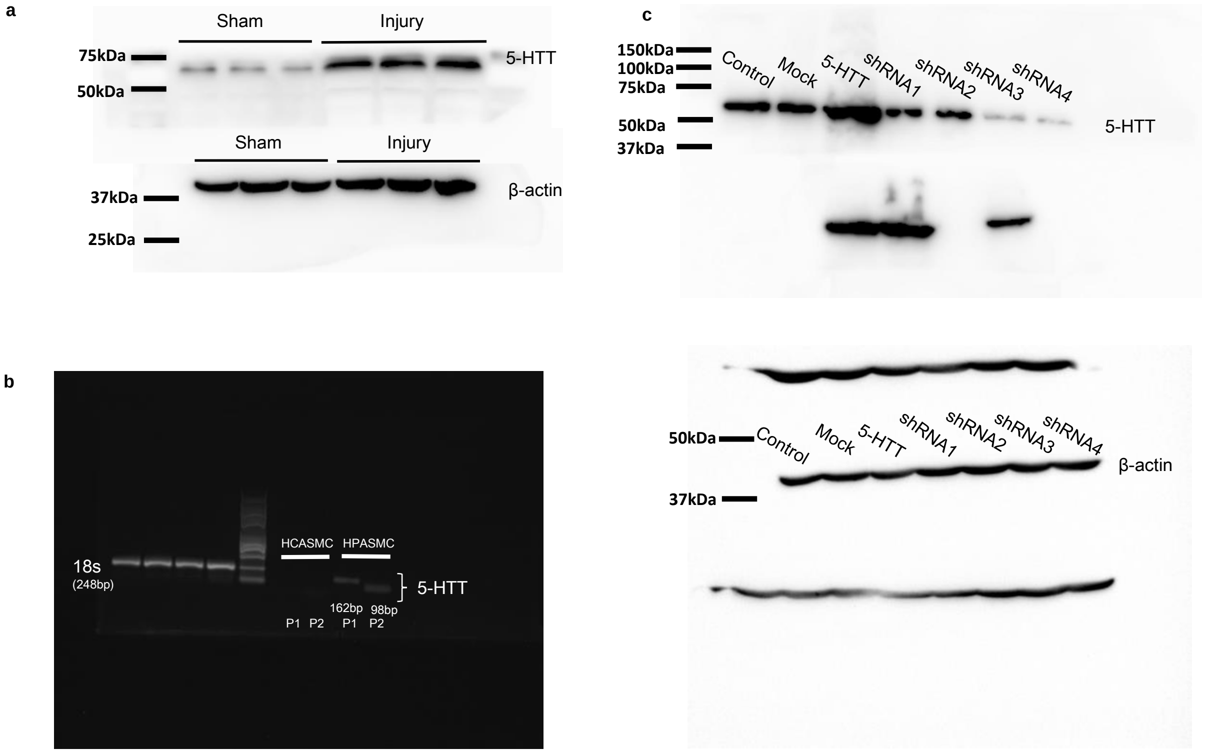

Supplementary Fig 1

(a) The protein level of 5-HTT in the injury and the normal carotid arteries from rat was measured by WB. (b) RT-PCR was used to detect the expression of 5-HTT in human coronary artery smooth muscle cell (HCASMCs) and human pulmonary artery smooth muscle cell (HPASMCs). P1: primer1, product=162bp; P2: primer2, product=98bp. (c) Western blot analysis confirmed the effects of 5HTT overexpression and shRNA knockdown on the protein levels compared to scrambled controls.

Supplementary Figure 2

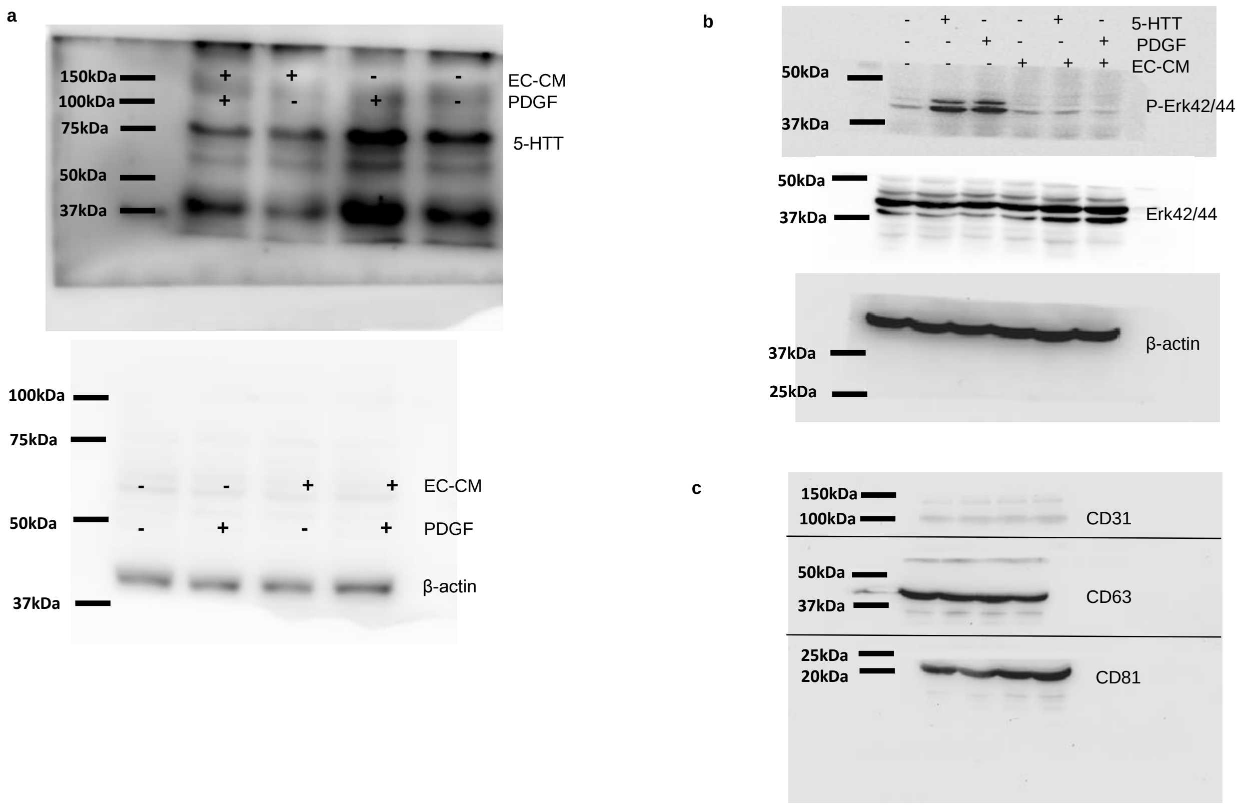

Supplementary Fig 2

(a) SMCs was cultured with EC-CM and the protein level of 5-HTT was analyzed by Western blot 48 hours later. (b)SMCs were incubated with EC-CM or PDGF for 48 hours. Phosphorylation of ERK was determined by Western blot analysis of the whole cell lysate using the phospho-specific antibody. (c)Western blot analysis of exosome protein.

Supplementary Figure 3

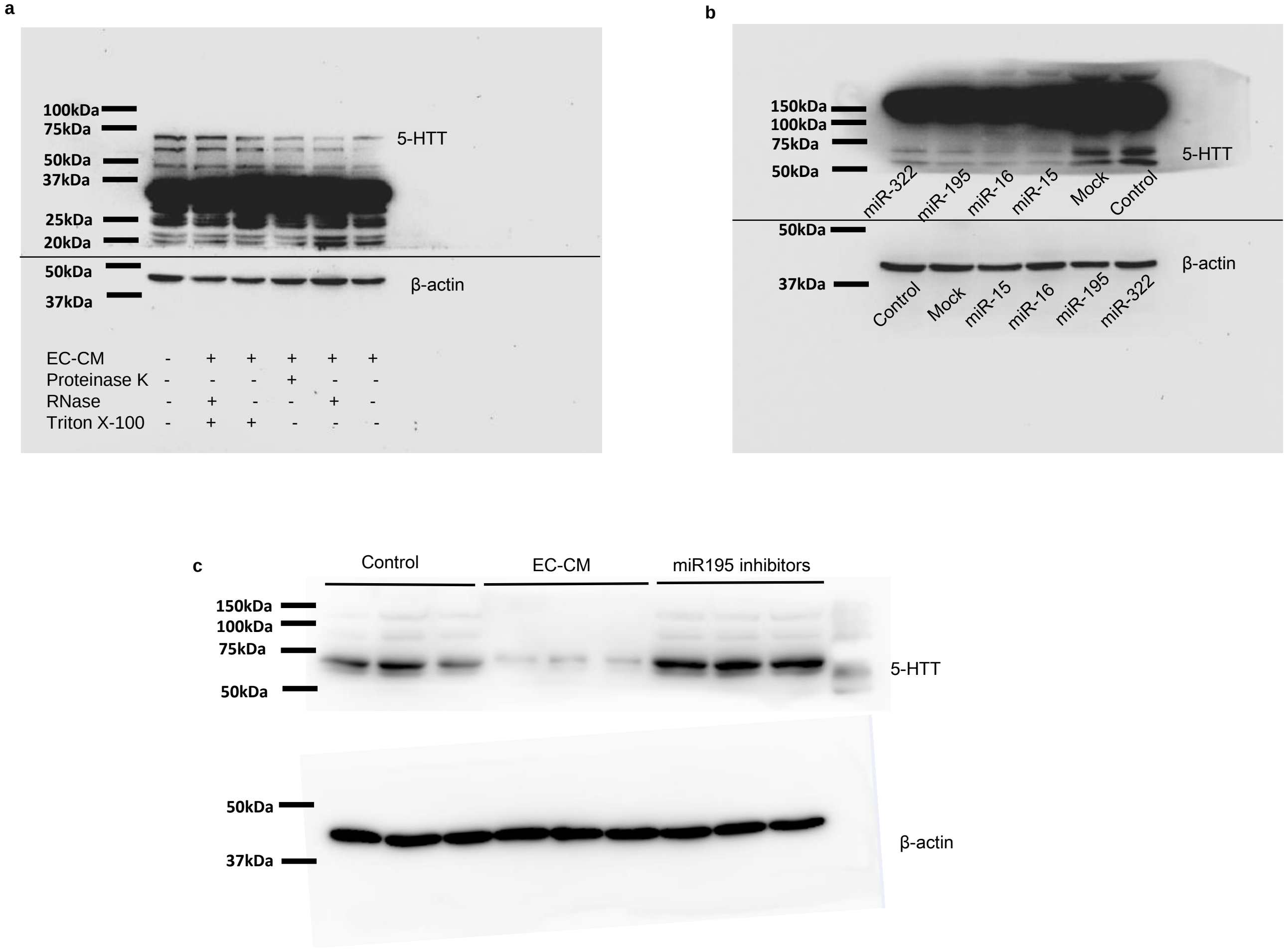

Supplementary Fig 3

(a) EC-CM was incubated with the indicated reagents cultured SMCs. The protein of SMCs was extracted 48 hours later and the levels of 5-HTT was measured by WB. (b) 5-HTT expression was inhibited after miR-15, miR-195, miR16, miR-322 mimics transfection in SMCs. The micro-RNA mimics were transfected into SMCs and the protein was extracted after 48 hours.
